# Comparative gonad transcriptome analysis in cobia (*Rachycentron canadum*)

**DOI:** 10.3389/fgene.2023.1128943

**Published:** 2023-04-06

**Authors:** Xueyan Shen, José M. Yáñez, Giana Bastos Gomes, Zhi Weng Josiah Poon, Derick Foster, Jorge F. Alarcon, Jose A. Domingos

**Affiliations:** ^1^ Tropical Futures Institute, James Cook University Singapore, Singapore, Singapore; ^2^ Facultad de Ciencias Veterinarias y Pecuarias, Universidad de Chile, Santiago, Chile; ^3^ Temasek Life Sciences Laboratory, National University of Singapore, Singapore, Singapore; ^4^ James Cook University, Singapore, Singapore; ^5^ Open Blue Sea Farms, Panama City, Panama; ^6^ Centre for Sustainable Tropical Fisheries and Aquaculture, James Cook University, Townsville, QLD, Australia

**Keywords:** cobia, gonad transcriptome, *Rachycentron canadum*, reproductive related pathways, sexual size dimorphism, sex-biased genes

## Abstract

**Background:** Cobia (*Rachycentron canadum*) is a species of fish with high commercial potential particularly due to fast growth rates. The evidence of sexual size dimorphism favoring females indicate potential benefits in having a monosex culture. However, the involvement of genetic factors responsible for sexual development and gonadal maintenance that produces phenotypic sex in cobia is largely unknown.

**Methods:** In the present study, we performed transcriptome sequencing of cobia to identify sex-biased significantly differentially expressed genes (DEGs) in testes and ovaries. The reliability of the gonad transcriptome data was validated by qPCR analysis of eight selected significantly differential expressed sex-related candidate genes.

**Results:** This comparative gonad transcriptomic analysis revealed that 7,120 and 4,628 DEGs are up-regulated in testes or ovaries, respectively. Further functional annotation analyses identified 76 important candidate genes involved in sex determination cascades or sex differentiation, including 42 known testis-biased DEGs (*dmrt1*, *amh* and *sox9 etc.*), and 34 known ovary-biased DEGs (*foxl2*, *sox3* and *cyp19a etc.*). Moreover, eleven significantly enriched pathways functionally related to sex determination and sex differentiation were identified, including Wnt signaling pathway, oocyte meiosis, the TGF-beta signaling pathway and MAPK signaling pathway.

**Conclusion:** This work represents the first comparative gonad transcriptome study in cobia. The putative sex-associated DEGs and pathways provide an important molecular basis for further investigation of cobia’s sex determination, gonadal development as well as potential control breeding of monosex female populations for a possible aquaculture setting.

## Introduction

Cobia (*Rachycentron canadum*) is a tropical aquaculture species with high potential due to fast growth rate, high flesh quality, high survival rates, and ease of spawning and larviculture (Holt et al., 2007). Exhibiting extreme growth rates reaching between 4 and 8 kg in the first year, cobia was first proposed as a potential aquaculture species by Hassler and Rainville (1975). Since then, cobia aquaculture has increased in popularity with global production rising from only 9 tonnes in 1997 to nearly 30,000 tonnes in 2007 (FAO, 2009), and reaching an estimated 55,000 tonnes in Taiwan, Panama, China, and Vietnam in 2019 (Tveterås et al., 2019). In recent years, research in cobia aquaculture has been focused on addressing knowledge gaps in commercial production, nutrition, and the use of genomic techniques to enhance its aquaculture performance. This is a response to its apparent slow growth in production during the 2010s (Tveterås et al., 2019; Benetti et al., 2021).

Cobia are multiple batch spawners, and reach sexual maturity within 2 years of age under aquaculture settings. The year-round spawning of large numbers of high-quality eggs with high fertilization has been shown possible through the control and manipulation of water parameters (Stieglitz et al., 2012). Cobia displays a strong sexual growth dimorphism with females growing significantly faster and reaching larger sizes than males, both in wild and captivity (Shaffer and Nakamura, 1989; Dutney et al., 2017; Molina et al., 2018; Díaz-Muñoz et al., 2019). Sexual size dimorphism creates the potential for a monosex female cobia population culture to maximise commercial production, similar to giant freshwater prawn (Nair et al., 2006; Levy et al., 2017) and Nile tilapia (Chakraborty et al., 2011; Moniruzzaman et al., 2015). However, the genetic mechanisms underlying cobia’s rapid growth rates, particularly in females, remains unknown, and to date there is no reliable method (i.e., sex-specific DNA markers) to distinguish the genotypic sex of cobia. Apart from limiting the potential advantage of a monosex female population culture, this also results in another major complication in cobia aquaculture: a breeding program with inefficient selection of broodstock where fast growing individuals, notably females, dominate the pool of breeding candidates when selection is based purely on weight. Without the ability to accurately test for the genotypic sex of cobia, breeding programs are unable to capitalise on potential genetic gains in desired traits necessary for commercial production such as fast growth, disease resistance, and survival (Benetti et al., 2021).

Sex determination mechanisms in fish are diverse and complex, ranging from genotypic sex determination (GSD) to environmental sex determination (ESD) systems. For most gonochoristic fish, the mechanism of genetic sex determination is associated with either polygene or a critical gene on sex chromosomes or autosomes (Kikuchi and Hamaguchi, 2013). Currently, many master sex determination genes have been reported to play a key role in regulating sex development in fish species [(for review see (Chen et al., 2022):], such as *dmy/dmrt1* in Japanese rice fish/medaka (*Oryzias latipes*) (Matsuda et al., 2002; Nanda et al., 2002), and *sdY* in rainbow trout (*Oncorhynchus mykiss*) (Yano et al., 2012)*.* These sex determining genes dictate the direction in development of bipotential gonads as either an ovary or a testis (Stévant and Nef, 2019). Cobia is considered a gonochoristic species as the even representation of males and females in its farmed cohorts, suggesting that there is a genetic system (i.e., sex chromosomes and a master gene) controlling the sex determination of the species (Shaffer and Nakamura, 1989). Unfortunately, cytologically there is no distinguishable sex chromosomes observed between genders (Jacobina et al., 2011; Benetti et al., 2021) in cobia. In addition, genomic information of the species is still scarce, and limited knowledge is known about the molecular mechanisms of sex determination and sex differentiation within cobia.

Transcriptome screening is one of the most powerful and efficient methods for discovering functional genes (Vidotto et al., 2013), as well as genetic markers. Gonads are indispensable reproductive organs, and their development is commonly controlled by multiple sex-associated genes and pathways. With the rapid development of sequencing technology, RNA sequencing (RNA-Seq) of gonadal samples has been used to examine sex determination or differentiation in various fish species (Lin et al., 2017; Fu et al., 2020; Li et al., 2020; Chen et al., 2021; Lin et al., 2021; Gao et al., 2022). The transcriptomic information available in cobia is currently limited. To date, only a liver transcriptome (pooled RNA samples extracted from hepatic tissue of ninety fish) (Barbosa et al., 2020) and some RNASeq data recently published identifying immune related genes in the liver, spleen and head kidney infected by *Photobacterium damselae subsp. Piscicida* (Tran et al., 2018) and *Streptococcus dysgalactiae* (Maekawa et al., 2019) are available. RNASeq has also been used to identify genes responsible for salinity regulation and adaptation in the gill of cobia (Cao et al., 2020; Huang et al., 2021). In the present study, we screened differentially expressed genes (DEGs) between testes and ovaries in cobia *via* comparative gonad transcriptome analysis, and identified sex-associated key genes and pathways. This work will offer a data source to further investigate the underlying molecular regulatory mechanisms of sex determination and gonadal development in cobia, which has a potential to contribute to a fast growing all-female population at a commercial level.

## Materials and methods

### Samples collection

**FIGURE 1:**
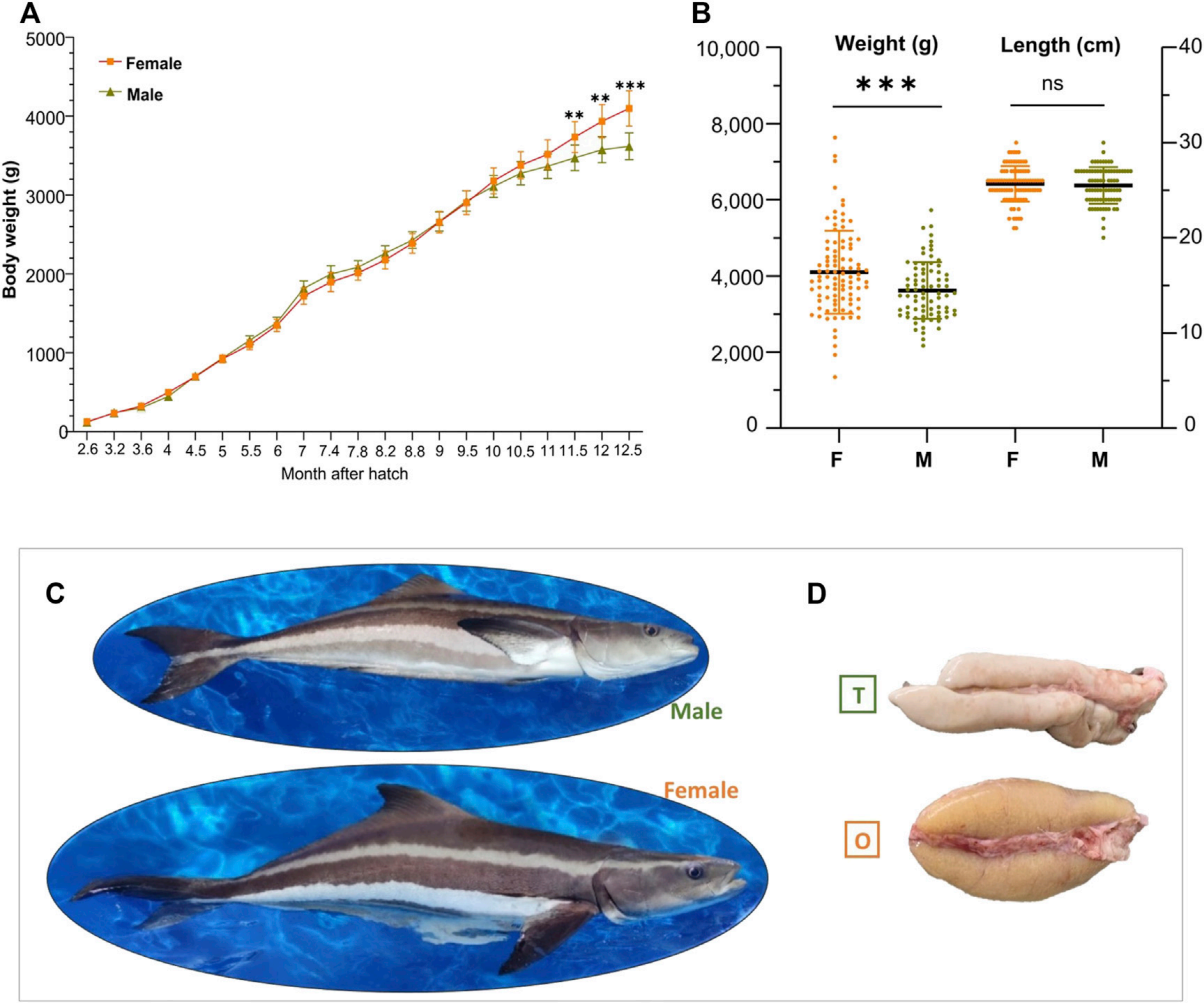
Sexual dimorphism in the growth of cobia. **(A)** Body weight of cobia at indicated time points after hatching (spawning date: 04/04/2018). **(B)** Body weight and body length of 1-year old adults (*n* = 170; M_Male_77&F_Female_93). “**”, *p < 0.01* and “***”, *p < 0.001*. **(C)** Representative photos of 2-year old male and female cobia. **(D)** Photos of the cobia gonads at adult stage. T, testis; O, ovary.

The gonad samples of cobia in this study were provided by Open Blue Sea Farms, the Republic of Panama. At the farm, the fish were cultured in 10 m^3^ circular HDPE tanks in flow through mode with incoming water treated by mechanical filtration, protein skimmer and UV filters. Water quality parameters were monitored daily, with averaged water temperatures of 27.5°C with range from 26°C in winter to 30°C in summer months, and also stable 35ppt full salinity water with pure oxygen injection through ceramic diffusers to maintain dissolved oxygen at 8 mg/L. In order to progressively track and determine if there is significant sexually dimorphic growth of male and female cobia, and especially the point of significant divergence in growth, the body weight and total length of 170 fish were individually PIT (Passive Integrated Transponder) tagged and their body weight were regularly weighted at twenty-one sampling points from an early age of ca. 3 months post hatching to 1 year old. The sex of individuals (77 males and 93 females) was determined through gonadal observations at the harvesting point and the gender fitted retrospectively to the growth data set. A total of twelve gonad tissues (six ovaries and six testes) for RNASeq were dissected from two-year-old adult fish of same cohort. After dissection, the determination of fish gender was performed by visual inspection of the gross morphology of gonads. The testes were clearly distinct from ovaries, and both sexes of the fish were at the spawning capable phase, late developing subphase (Lefebvre and Denson, 2012). Ovaries were observed to be well developed with vitelogenic eggs clearly visible and milt was present in testes (Figure 1D). All the samples were placed in RNAlater (Ambion) and stored at −80°C before RNA isolation.

### RNA isolation, library construction and sequencing

Total RNA was extracted from each gonad tissue using RNeasy^®^ Mini kit (Qiagen, 74104), following the manufacturer’s instructions. All RNA samples were treated with RNase free DNase-I (M610A, Promega) to remove genomic DNA contamination. The quality and the quantity of the total RNA was determined with an Agilent 2100 Bioanalyzer (RNA 6000 Nano chip assay) and a Qubit 3.0 (Quant-It dsRNA BR Assay). Only the RNA samples with RNA integrity number (RIN) > 8.5 were used for constructing the cDNA library in HiSeq. A total of ten libraries of gonadal samples (one testis and one ovary not included for library construction due to the poor quality of RNA) were generated using VAHTS mRNASeq V3 Library Prep Kit for Illumina (NR611, Vazyme), following manufacturer’s instructions. Briefly, mRNA with poly(A) was enriched by mRNA Capture Beads and fragmented by heating. Short mRNA was reverse-transcribed with random hexamer-primers to generate the first cDNA, and then the second cDNA was synthesized. cDNA fragments went through an end repair process, the addition of a single ‘A’ base to the 3’ end and then ligation of the adapters. Then the products were purified and size selected (350 bp range). At the end, fragments were enriched by PCR amplification and purified using VAHTSTM DNA Clean Beads. The quality and quantity of the PCR product was determined by the Agilent Bioanalyzer 2100 and Qubit 2.0 (Thermo). Finally, ten cobia sequencing libraries (five from testes and five from ovaries with RIN value >8.5) were sequenced on an Illumina Novaseq 6000 platform with 150 bp paired-end reads.

### Sex-biased gene differential expression, functional GO and pathway enrichment analysis

High-quality clean data were produced from the raw data by removing reads containing adapters, more than 10 unknown nucleotides, or more than 50 low-quality (Q ≤ 20) bases. Paired-end clean reads were aligned with the cobia reference genome (unpublished data) using TopHat v2.0.12 (Kim et al., 2013). Genetic quantification of gene expression level was determined with HTSeq v0.6.1 by counting the reads numbers mapped to each gene (Anders et al., 2015). The expected number of fragments per kilobase of transcript sequence per million base pairs sequenced (FPKM) of each gene was calculated based on the length of the gene and reads count mapped to this gene. To characterize differentially expressed genes (DEGs) between ovary and testis, the raw reads number data sets were analyzed using the DESeq R package (1.18.0) (Wang et al., 2009). Genes with an adjusted *p*-value <0.05 and |log2FoldChange|≥0.7 were assigned as the threshold for indicating significant differential expression. Gene Ontology (GO) enrichment analysis of differentially expressed genes was implemented by the GOseq R package, in which gene length bias was corrected (Young et al., 2010). GO terms with corrected *p*-values *< 0.05* were considered significantly enriched by differentially expressed genes. KOBAS software (Mao et al., 2005) was utilized to test the statistical enrichment of those differential expression genes in Kyoto Encyclopedia of Genes and Genomes (KEGG, http://www.genome.jp/kegg/) pathways (Kanehisa et al., 2016). The protein-protein interaction (PPI) network prediction (http://cn.string-db.org) was adopted for a set of selected candidate sex-related DEGs (Minimum required interaction score was 0.4.) and the obtained gene target network was imported into Cytoscape software (https://cytoscape.org/) for visual editing.

### Validation of the DEGs by quantitative real-time PCR (qPCR)

The reliability of the gonad transcriptome data was validated by qPCR analysis of eight selected significantly differentially expressed sex-related candidate genes. The primers were designed using Primer3 (Rozen and Skaletsky, 2000) on the Geneious Prime 2021.2.2 (Biomatters) software. Total RNA was isolated from gonad samples of three males and three females using RNeasy^®^ Plus Micro Kit (Qiagen) according to the manufacturer’s instructions. The RNA was then subjected to reverse transcription using a SensiFAST cDNA Synthesis Kit (Bioline). qPCR was performed on StepOnePlus™ Real-Time PCR System thermal cycler (Applied Biosystems) using KAPA SYBR FAST qPCR Master Mix (2X) (Sigma-Aldrich). Gene expression stability of all samples for *ubq*, *ef1a*, and *b-actin* was analysed using the geNorm (Vandesompele et al., 2002), plugin on the qbase+ 3.3 software (Biogazelle), of which, *ubq* was determined to be the most stable (M = 1.46), and was chosen as an internal control to determine relative expression. The relative gene expression levels were calculated using the 2^−ΔΔCt^ method (Livak and Schmittgen, 2001), normalised by *ubq*. All statistical analysis between testes and ovaries were performed on SPSS Statistics v28.0.1.1 (IBM). Values with *p < 0.05* were considered significant.

## Results

### Sexually dimorphic growth in cobia

To characterize the sexual dimorphism in growth of cobia, fish of both sexes were measured at twenty-one sampling points from ca. 3 months post hatching. As shown in Figure 1A, female-biased growth was observed after 9.5 months of age (avg. weight of M_2.93 kg; F_2.90 kg), with differences in weight between the sexes increasing as the trial continued. However, statistical differences were only observed from 11.5 months of age. At the end of the experiment, female fish were significantly (*p < 0.001*) heavier than males (13.5%), with an average body weight of 4.11 kg compared to 3.62 kg, for females and males, respectively (Figure 1B). The females also had longer average body length compared to the males although no significant difference was observed (Figures 1B, C). These results confirm that female cobia have higher growth rates and larger size at maturation than males. Figure 1D shows that testis is clearly distinct from ovary in the species at adult stage.

### Overview of sequencing data and read mapping of testis and ovaries

RNASeq of all ten libraries produced 488,232,862 (73.23 Gb) and 601,664,668 (90.25 Gb) raw reads from testes and ovaries, respectively (Supplementary Table S1). All raw sequencing data were submitted to the Sequence Read Archive (SRA) of the NCBI database under BioProject accession number PRJNA853560. After removing low-quality reads, rRNA reads, reads containing adapters, and reads with >10 unknown nucleotides, 485,824,650 (72.87 Gb) and 598,371,962 (89.76 Gb) clean reads were remained for testis and ovaries, respectively. An average of 16.26 Gb (ranging from 11.55 to 37.95 Gb) clean reads were generated from each sample. The Q20 and Q30 ranged from 96.55% to 98.26% and 92.52%–94.93% respectively, indicating that the sequencing data were of high quality. All the filtered clean reads were then mapped individually against the annotated reference genome of a male cobia (unpublished data), with an averaged mapping rate of 83.76 per sample (Supplementary Table S1).

### Identification and enrichment analysis of the differentially expressed genes (DEGs) in testes and ovaries of cobia

**FIGURE 2:**
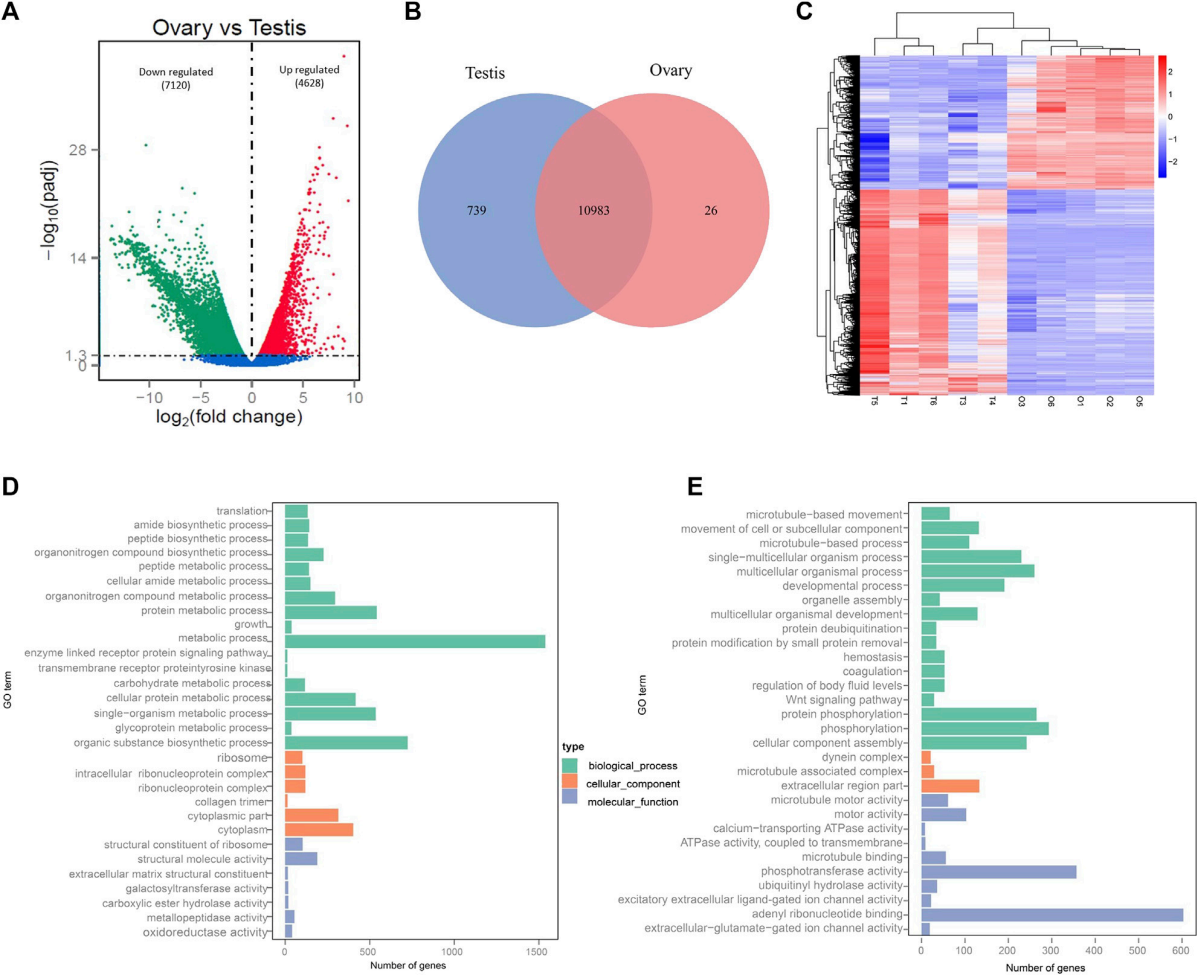
Identification and GO functional annotation of DEGs between testes and ovaries. **(A)** Number of up-/down-expressed DEGs in testes versus ovaries. **(B)** Venn diagram of testis-specific and ovarian-specific genes. **(C)** Heatmap analysis of hierarchical clustering of DEGs in males (T1, T3, T4, T5, T6) and females (O1, O2, O3, O5, O6). Each column represents an individual, and each row represents a gene. Blue and red colors in the heatmap correspond to low and high relative gene expression, respectively. **(D)** Top 30 enriched GO terms for ovary-biased DEGs. **(E)** Top 30 enriched GO terms for testis-biased DEGs.

By comparison of the unigene expression levels in gonadal transcriptomes, a total of 11,748 DEGs were detected between the two sexes (Supplementary Table S2). Compared to the testes, there were 4,628 upregulated DEGs and 7,120 downregulated DEGs in the ovaries (Figure 2A). Further analysis indicated that the number of male-specific expressed genes was larger than those of females (739 vs. 26) (Figure 2B; Supplementary Table S2). In addition, the heatmap generated based on all the DEGs detected in ovaries and testes showed the transcriptomic profiles of testes was obviously different than the ovaries (Figure 2C).

**FIGURE 3:**
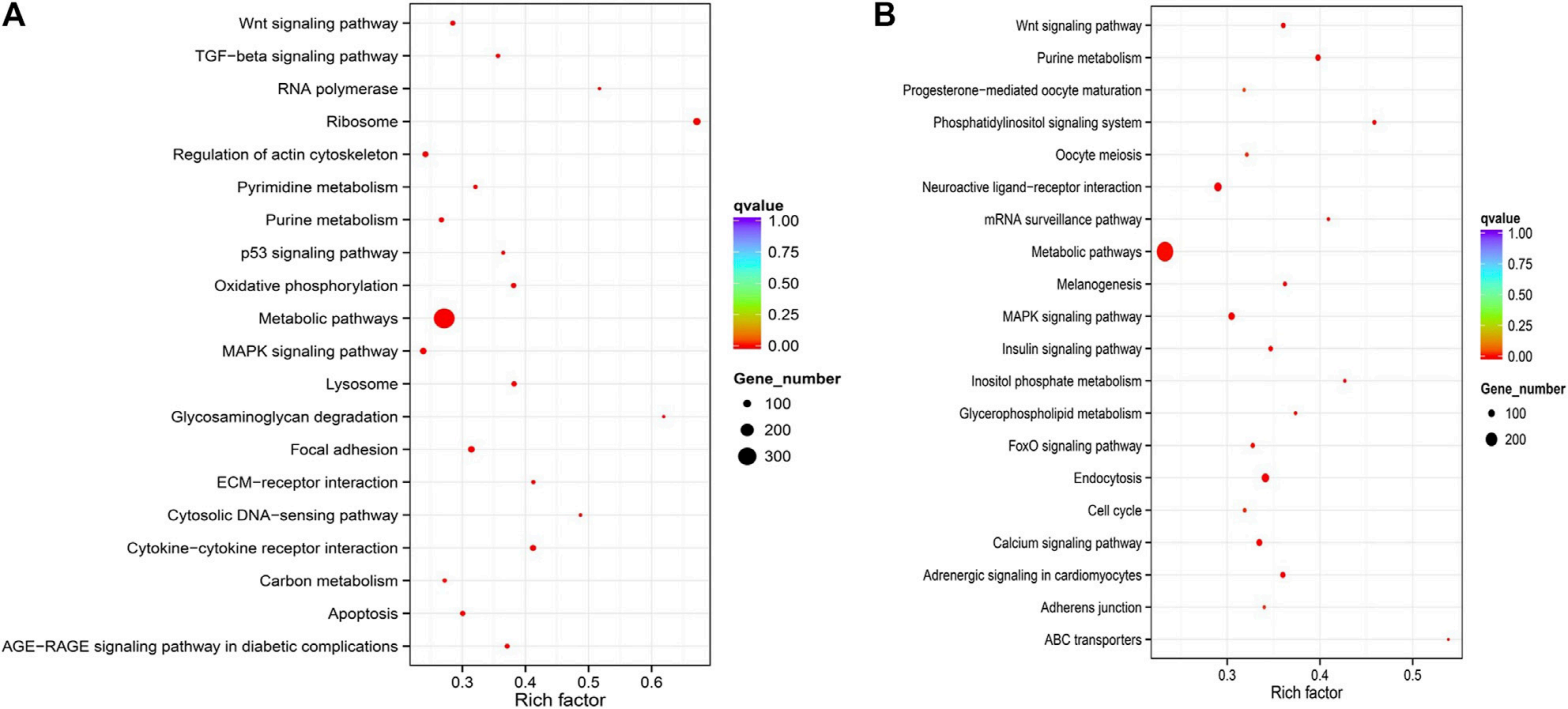
Top20 KEGG pathways enrichment of upregulated DEGs (Ovary vs Testis) **(A)** and downregulated DEGs (Ovary vs Testis) **(B)**.

Furthermore, GO functional annotation and KEGG pathway analysis were searched for all DEGs. A total of 8,543 unigenes (5,237 female-biased genes and 3,306 male-biased genes) were assigned to 4,265 GO terms (Supplementary Table S3). The DEGs were primarily classified within “biological processes”, followed by “molecular function” and then “cellular component”. More importantly, many DEGs were involved with sex-related GO terms, such as gonad development, gamete generation, germ cell development, sexual reproduction, sex differentiation, spermatogenesis, reproductive processes and meiotic cell cycle processes, etc. (Supplementary Table S3). In addition, the GO annotation of ovary-biased DEGs (Figure 2D; Supplementary Table S4) showed that the top three most significant GO terms were structural constituent of ribosome, ribosome and translation; while microtubule-based movement, microtubule motor activity and movement of cell or subcellular were the top three most significant GO terms for testis-biased DEGs (Figure 2E; Supplementary Table S5). KEGG pathway enrichment analysis indicated that the DEGs were annotated in 151 signaling pathways (Supplementary Table S6). Figure 3 shows the top 20 most significantly enriched pathways (*p* < 0.05) for ovary upregulated (Figure 3A) and downregulated DEGs (Figure 3B), respectively. Notably, eleven out of these top significantly enriched pathways functionally related to reproduction, sex determination and differentiation were identified, including Wnt signaling pathway, TGF-beta signaling pathway, MAPK signaling pathway and p53 signaling pathway (Supplementary Table S7) associated with female-biased DEGs; as well as endocytosis, adrenergic signaling in cardiomyocytes, neuroactive ligand-receptor interaction, Wnt signaling pathway, MAPK signaling pathway, FoxO signaling pathway, Insulin signaling pathway, oocyte meiosis and progesterone-mediated oocyte maturation associated with male-biased DEGs (Supplementary Table S8).

### Analysis of the expression pattern of representative gonadal genes implicated in sex determination and sex differentiation of cobia

**FIGURE 4:**
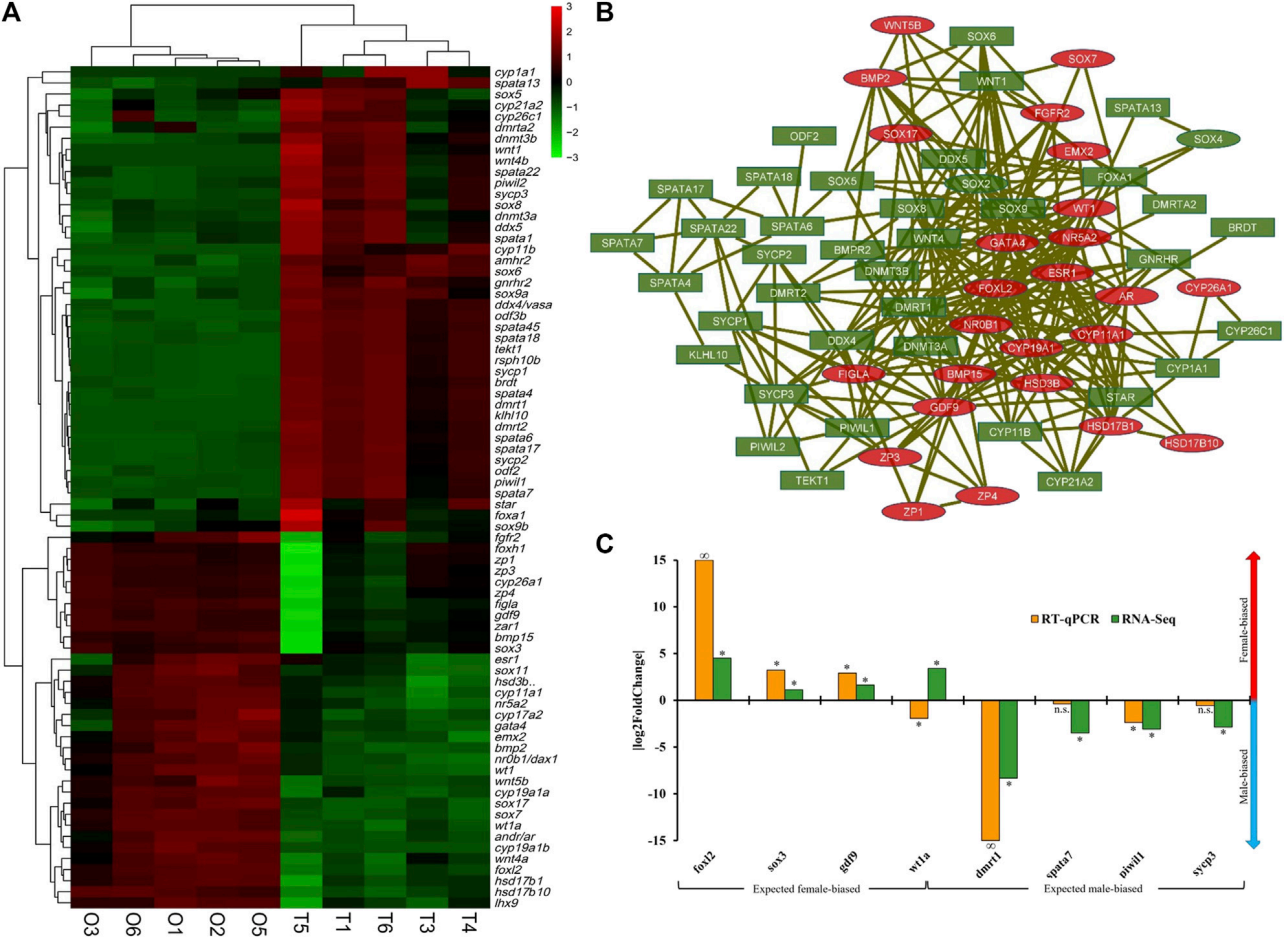
Sexually differentially expressed genes (DEGs) of cobia (*p* < 0.05). **(A)** The expression pattern of the representative gonadal genes implicated in sex determination/differentiation of cobia. In the heatmap, each row represents a gene listed on the right. Each column stands for a gonadal sample of males (T1, T3, T4, T5, T6) and females (O1, O2, O3, O5, O6). The expression of genes is colour coded from low (green) to high (red). M: male-biased, F: female-biased. **(B)** The testis upregulated (green with rectangle shapes) and ovary upregulated sex candidate genes (red with oval shapes) were filtered into the DEG PPI network complex. Nodes represent genes, edges represent correlation between nodes. **(C)** Validation of the expression patterns of selected significantly expressed DEGs from RNA-Seq, using qPCR. Positive |log2FoldChange| indicates female-biasness, negative values indicate male-biasness. ‘*’represents significant difference between testes and ovaries (*p-value < 0.05*), n.s. Represents non-significance, ‘∞’ represents infinite log2FoldChange for RT-qPCR results.

Based on the resources of annotation and enrichment analyses, the DEGs were further comparatively analysed. A total of seventy-six either well known candidate gonadal sex differentiation genes or genes involved in the sex determination cascade of teleosts were detected in the cobia gonadal transcriptome, which showed significant sexually dimorphic expression between the testes and ovaries (Table 1). Among them, forty-two were upregulated in the testes, while thirty-four exhibited significantly higher expressions in the ovaries (Table 1; Figure 4A). The interaction networks of the DEGs are shown in Figure 4B. The genes essential for testis differentiation and maintenance of male specified germ cells in fish included: doublesex and mab-3 related transcription factor genes (*dmrt1*), anti-Müllerian hormone (*amh*) and its receptor *amhr2*, SRY (sex-determining region Y)-box genes (*sox9a*, *sox9b*, *sox8*), piwi-like protein genes (*piwil1* and *piwil2*), cytochromes P450 enzyme (*cyp11b, cyp17a2*), which were identified and significantly upregulated in the testes of cobia. The genes associated with spermatogenesis such as synaptonemal complex protein (*sycp1, sycp2, sycp3*), Kelch-like protein 10 (*klhl10*), tekt1 (*Tektin-1*), spermatogenesis-associated genes (*spata1, spata6, spata13, spata17*), outer dense fiber protein (*odf3*, *odf3b* and *odf2*), DEAD-box helicase (Ddx) family members (*ddx4/vasa, ddx5*), and others were also highly upregulated in testes. The expressions of genes related to ovary differentiation including: *sox3, sox7, sox11, sox17*, forkhead transcription factor L2 gene (*foxl2*), cytochromes P450 enzyme (*cyp19a1, cyp11a1*), factor in the germline alpha (*figla*), GATA Binding Protein 4 (*gata4*), Nuclear receptors superfamily genes (*nr5a2, nr0b1/dax1*)), Wnt signaling components (*wnt4a, wnt5b*), hydroxysteroid dehydrogenases (*hsd17b1, hsd17b10, hsd3b*), zona pellucida sperm-binding proteins (*zp1, zp3, zp4*), Wilm’s tumor 1 (*wt1*), gonadal soma derived factor 1 (*gdf9*) and bone morphogenetic protein (*bmp2, bmp15*), were highly upregulated in ovaries. Among these seventy-six representative reproductive related DEGs, we found that two cyp genes of *cyp11b and cyp1a1,* as well as two wnt family members, *wnt1* and *wnt4b,* showed male-specific expression patterns.

**TABLE 1:** Candidate sex-associated DEGs identified in the gonad of Cobia (“*na.”*: male-specific).

Gene name	Gene description	log2FoldChange (Ovary/Testis)	Padj	Sex-bias	Gene ID
*cyp1a1*	Cytochrome P450 Family 1 Subfamily A Member 1	*na*.	0.00232	Male	cobia_male_GLEAN_10015121
*spata13*	Spermatogenesis-associated protein 13	−1.86	0.02685	Male	cobia_male_GLEAN_10003586
*sox5*	SRY-box containing protein 5	−1.43	0.01219	Male	cobia_male_GLEAN_10002305
*cyp21a2*	Cytochrome P450 family 21 subfamily a, polypeptide 2	−1.60	0.01390	Male	cobia_male_GLEAN_10016468
*cyp26c1*	Cytochrome P450, family 26, subfamily c, polypeptide 1	−1.60	0.01577	Male	cobia_male_GLEAN_10010878
*dmrta2*	Doublesex- and mab-3-related transcription factor A2	−1.46	0.01520	Male	cobia_male_GLEAN_10013443
*dnmt3b*	DNA methyltransferase 3b	−9.69	3.1843E-13	Male	cobia_male_GLEAN_10017642
*wnt1*	Wnt Family Member 1	*na.*	1.3726E-06	Male	cobia_male_GLEAN_10018125
*wnt4b*	Wnt Family Member 4	*na.*	3.692E-08	Male	cobia_male_GLEAN_10015723
*spata22*	Spermatogenesis-associated protein 22	−1.88	0.00146	Male	cobia_male_GLEAN_10009981
*piwil2*	Piwi-like protein 2	−1.61	0.00079	Male	cobia_male_GLEAN_10015240
*sycp3*	Synaptonemal complex protein 3	−2.86	0.00016	Male	cobia_male_GLEAN_10000494
*sox8*	SRY-box containing protein 8	−3.13	0.00019	Male	cobia_male_GLEAN_10005650
*dnmt3a*	DNA methyltransferase 3a	−2.49	0.00026	Male	cobia_male_GLEAN_10005336
*ddx5*	DEAD-box helicase 5	−1.86	0.00072	Male	cobia_male_GLEAN_10019286
*spata1*	Spermatogenesis-associated protein 1	−1.99	0.00021	Male	cobia_male_GLEAN_10013527
*cyp11b*	Cytochrome P450 family 11 subfamily b	*na.*	2.6019E-11	Male	cobia_male_GLEAN_10015619
*amhr2*	Anti-mullerian hormone type 2 receptor	−2.52	3.6482E-06	Male	cobia_male_GLEAN_10019873
*sox6*	SRY-box containing protein 6	−4.94	2.071E-08	Male	cobia_male_GLEAN_10017396
*gnrhr2*	Gonadotropin Releasing Hormone Receptor 2	−3.19	3.8631E-07	Male	cobia_male_GLEAN_10016097
*sox9a*	SRY-box containing protein 9	−2.18	4.1361E-05	Male	cobia_male_GLEAN_10002612
*ddx4/vasa*	Probable ATP-dependent RNA helicase DDX4	−3.79	3.1148E-11	Male	cobia_male_GLEAN_10001182
*odf3b*	Outer dense fiber protein 3	−12.13	1.3197E-15	Male	cobia_male_GLEAN_10016959
*spata45*	Spermatogenesis-associated protein 45	−9.00	8.8769E-11	Male	cobia_male_GLEAN_10000464
*spata18*	Spermatogenesis-associated protein 18	−9.56	1.8203E-12	Male	cobia_male_GLEAN_10013097
*tekt1*	Tektin-1	−9.21	1.7851E-12	Male	cobia_male_GLEAN_10012654
*rsph10b*	Radial Spoke Head 10 Homolog B	−11.40	2.1882E-16	Male	cobia_male_GLEAN_10002701
*sycp1*	Synaptonemal complex protein 1	−11.39	1.7912E-17	Male	cobia_male_GLEAN_10019801
*brdt*	Bromodomain testis-specific protein	−5.53	1.239E-07	Male	cobia_male_GLEAN_10013185
*spata4*	Spermatogenesis-associated protein 4	−10.51	1.2068E-13	Male	cobia_male_GLEAN_10014296
*dmrt1*	Doublesex and mab-3 related transcription factor 1	−8.31	1.5076E-10	Male	cobia_male_GLEAN_10014514
*klhl10*	Kelch-Like Protein 10	−13.35	3.4662E-15	Male	cobia_male_GLEAN_10006974
*dmrt2*	Doublesex and mab-3 related transcription factor 2	−7.56	1.3688E-09	Male	cobia_male_GLEAN_10014515
*spata6*	Spermatogenesis Associated 6	−5.84	8.9617E-08	Male	cobia_male_GLEAN_10013438
*spata17*	Spermatogenesis-associated protein 17	−5.47	1.5111E-07	Male	cobia_male_GLEAN_10020965
*sycp2*	Synaptonemal complex protein 2	−5.30	2.1906E-07	Male	cobia_male_GLEAN_10018260
*odf2*	Outer dense fiber protein 2	−4.62	1.5014E-06	Male	cobia_male_GLEAN_10014488
*piwil1*	Piwi-like protein 1	−3.08	1.2167E-05	Male	cobia_male_GLEAN_10015178
*spata7*	Spermatogenesis-associated protein 7	−3.51	2.5786E-05	Male	cobia_male_GLEAN_10008788
*star*	Steroidogenic acute regulatory protein	−1.84	0.02895	Male	cobia_male_GLEAN_10015137
*foxa1*	Forkhead Box A1	−4.06	0.02008	Male	cobia_male_GLEAN_10008402
*sox9b*	SRY-box containing protein 9b	−1.42	0.01441	Male	cobia_male_GLEAN_10000422
*fgfr2*	DANRE Fibroblast growth factor receptor 2	0.94	0.04620	Female	cobia_male_GLEAN_10010412
*foxh1*	Forkhead transcription factor H1	0.91	0.03010	Female	cobia_male_GLEAN_10007129
*zp1*	zona pellucida sperm-binding protein	1.34	0.00320	Female	cobia_male_GLEAN_10007933
*zp3*	zona pellucida sperm-binding protein 3	1.17	0.00228	Female	cobia_male_GLEAN_10007900
*cyp26a1*	Cytochrome P450 Family 26 Subfamily A Member 1	1.10	0.00351	Female	cobia_male_GLEAN_10002507
*zp4*	Zona pellucida sperm-binding protein 4	1.82	0.00010	Female	cobia_male_GLEAN_10006970
*figla*	Factor in the germline alpha	1.43	0.00090	Female	cobia_male_GLEAN_10015270
*gdf9*	Growth differentiation factor 9	1.64	0.00013	Female	cobia_male_GLEAN_10013979
*zar1*	zygote arrest protein 1	2.09	1.8135E-06	Female	cobia_male_GLEAN_10009482
*bmp15*	Bone morphogenetic protein	1.25	0.00355	Female	cobia_male_GLEAN_10001399
*sox3*	SRY-box containing protein 3	1.14	0.00753	Female	cobia_male_GLEAN_10013735
*esr1*	Estrogen Receptor 1	1.23	0.04104	Female	cobia_male_GLEAN_10005155
*sox11*	SRY-box containing protein 11	1.35	0.02385	Female	cobia_male_GLEAN_10016356
*hsd3b*	3 beta-hydroxysteroid dehydrogenase	2.56	1.3255E-06	Female	cobia_male_GLEAN_10011123
*cyp11a1*	Cytochrome P450 Family 11 Subfamily A Member 1	2.55	1.8978E-06	Female	cobia_male_GLEAN_10017092
*nr5a2*	Nuclear receptor subfamily 5 group A member 2	2.65	7.2504E-07	Female	cobia_male_GLEAN_10015331
*cyp17a2*	17-alpha-hydroxylase	2.07	0.00247	Female	Novel02696
*gata4*	GATA Binding Protein 4	1.82	0.00130	Female	cobia_male_GLEAN_10005096
*emx2*	Empty Spiracles Homeobox 2	1.21	0.02099	Female	cobia_male_GLEAN_10010277
*bmp2*	bone morphogenetic protein 2	3.21	6.4924E-05	Female	cobia_male_GLEAN_10004919
*nr0b1/dax1*	(Nuclear Receptor Subfamily 0 Group B Member 1	2.92	2.6934E-08	Female	cobia_male_GLEAN_10011261
*wt1*	Wilms tumor protein 1a	3.04	8.0628E-09	Female	cobia_male_GLEAN_10016690
*wnt5b*	Wnt Family Member 5B	1.93	0.00030	Female	cobia_male_GLEAN_10002192
*cyp19a1a*	cytochrome P450, family 19, subfamily A, polypeptide 1a	4.29	1.7254E-18	Female	cobia_male_GLEAN_10021342
*sox17*	SRY-box containing protein 17alpha	2.82	1.0226E-07	Female	cobia_male_GLEAN_10003736
*sox7*	SRY-box containing protein 7	4.48	5.6352E-17	Female	cobia_male_GLEAN_10005057
*wt1a*	Wilms tumor protein a	3.42	5.8198E-12	Female	cobia_male_GLEAN_10021031
*andr/ar*	Spermatogenesis-associated protein 13	3.04	1.845E-09	Female	cobia_male_GLEAN_10009917
*cyp19a1b*	cytochrome P450, family 19, subfamily A, polypeptide 1b	8.24	4.6176E-25	Female	cobia_male_GLEAN_10017169
*wnt4a*	Wingless-type MMTV integration site family, member 4a	2.05	2.3331E-05	Female	cobia_male_GLEAN_10020551
*foxl2*	Forkhead transcription factor L2	4.52	3.1122E-18	Female	cobia_male_GLEAN_10012619
*hsd17b1*	Estradiol 17-beta-dehydrogenase 1	3.18	1.0598E-10	Female	cobia_male_GLEAN_10005849
*hsd17b10*	Hydroxysteroid 17-Beta Dehydrogenase 10	1.93	1.5469E-05	Female	cobia_male_GLEAN_10017620
*lhx9*	LIM/homeobox protein Lhx9	1.74	0.00023	Female	cobia_male_GLEAN_10012948

### Verification of DEGs by qPCR

To validate the transcriptome data, expression profiles of four selected male-biased genes (*dmrt1, spata7, piwil1, sycp3*) and four female-biased genes (*sox3, gdf9, foxl2, wt1a*) representing sex-related candidate genes were individually evaluated by qPCR (primer sequences in Supplementary Table S9). The results showed that except for *wt1a,* the overall up- or downregulation expression profiles of all four male-biased DEGs and three female-biased DEGs were consistent between qPCR and transcriptome data (Figure 4C), indicating the validity of the RNASeq data. Interestingly, two genes showed sex-specific expression with *foxl2* detected only in female and *dmrt1* only in male of cobia individuals by qPCR (Figure 4C).

## Discussion

This study revealed previous findings (Shaffer and Nakamura, 1989; Dutney et al., 2017; Molina et al., 2018; Díaz-Muñoz et al., 2019) that female cobia grow significantly faster than male fish. Sexual size dimorphism has been observed in a number of commercially significant fish species [for review see (Mei and Gui, 2015):]. For sexual maturity of cobia, males (around 1 year old) have a lower average age than females (1.5–2 year old) (Kuang et al., 2021). In this study, the divergence in growth rate between male and female fish at 11.5 months post-hatching seems to start around the age of male maturation. The earlier maturation in males is usually associated with slower growth rates as more energy investment into reproduction (Hüssy et al., 2012). The potential mechanism for the divergence in sexual size dimorphism phenomena of cobia needs to be further investigated.

The current study represents the first comparative gonad transcriptome studies of cobia to date. The identified sexually dimorphic genes (DEGs), especially the seventy-six well documented candidate genes associated with sex determination and sex-specific gonad development may be effective indicators for cobia genotypic sex prediction; such as the DM-domain-containing genes of *dmrt1, dmrt2* and *dmrta2. dmrt1* is essential for the maintenance of male-specified germ cells and testes differentiation (Webster et al., 2017). In fish, *dmrt1* is a master sex-determining gene in half-smooth tongue sole (*Cynoglossus semilaevis*) (Cui et al., 2017), and its duplicated copy of *dmy* was confirmed as a sex-determining gene in medaka (*Oryzias latipes*) (Matsuda et al., 2002; Bratuś and Słota, 2006; Herpin and Schartl, 2011). In the protandrous hermaphrodite barramundi (*Lates calcarifer*) (Domingos et al., 2018), higher methylation levels in the promoter region of *dmrt1* in female gonads is associated with the total splicing out of DM domain in *dmrt1* mRNA, indicating a seemingly ubiquitous sex related role of this transcription factor in vertebrates. In the present study, *dmrt1* was highly expressed in the testes of cobia (log2FC = 8.3). The trend of its expression was quite similar to those in other fish such as tilapia (*O. niloticus*) (Kobayashi and Nagahama, 2009; Tao et al., 2013), rainbow trout (Cavileer et al., 2009) and blunt snout bream (*Megalobrama amblycephala*) (Su et al., 2015), suggesting that *dmrt1* may be a key player for cobia testicular differentiation. In addition, the *dmrt2* gene highly expressed in male germ cells in Chinese tongue sole (Zhu et al., 2019), was also significantly upregulated during ovary-to-testis sex reversal in the swamp eel (Sheng et al., 2014). Here, *dmrt2* was dominantly expressed in male gonads (log2FC = 7.6) of cobia, indicating this gene may play a functional role in gonadal differentiation/development and germ cell maturation in the testes of cobia.

The present study also detected eight members of the SOX gene family including *sox9*, *sox8*, *sox6* and *sox5* highly expressed in testes, whereas the *sox3*, *sox7*, *sox11* and *sox17* were dominantly expressed in ovaries, suggesting that the SOX genes play complicated roles in sex differentiation in cobia. The sex-determining region Y (*SRY*) is a sex determining gene in humans (Maloy and Hughes, 2013), which is the key switch for individuals developing into males or females. Several other *SRY*-related HMG-box transcription factors were also found to be involved in the regulation of sex determination and differentiation in fish. *sox3* is the male-determining factor on the Y chromosome in the fish *Oryzias dancena* (Takehana et al., 2014), while it was found mainly expressed in the ovaries of yellowfin seabream (*Acanthopagrus latus*) (Li et al., 2020). In addition, *sox2* is the master sex determination gene in turbot (Martínez et al., 2019). Notably, the *sox9* gene, a major transcription factor in testicular development, has been widely studied in various fish species, for example, rainbow trout and Japanese flounder (*Paralichthys olivaceus*) (Baron et al., 2005; Raghuveer and Senthilkumaran, 2010; Li et al., 2018).

The forkhead-box L2 (*foxl2*) transcription factor is considered a marker of ovarian differentiation in vertebrates including fish. As in gonochoristic fish species, *foxl2* was exclusively expressed in somatic cells of developing (pre-vitellogenic) ovaries (Wang et al., 2004; Nakamoto et al., 2006; Yamaguchi et al., 2007; Nakamoto et al., 2009; Jiang et al., 2011). In protogynous hermaphrodite fish, *foxl2* expression level declines from early ovarian developmental stages to the stage of transition to testes (Kobayashi et al., 2010; Hu et al., 2014a), and *vice versa* in protandrous species (Wu and Chang, 2009). In our study, *foxl2* was predominantly expressed in females (log2FC = 4.5), suggesting it is associated with female gonad differentiation in cobia. Also, *foxl2* functions by binding to the promoter region of *cyp19a* (P450 aromatase) activating transcription and regulating estrogen synthesis (Zhang et al., 2017). In fish, *cyp19a1* plays a pivotal role in sex differentiation and ovary development by converting testosterone into estradiol, and is regarded as a reliable early marker of ovarian differentiation (Yuan et al., 2021). In the present study, *cyp19a1* was found to be highly upregulated in ovaries compared to testes, exhibiting a similar expression pattern to other fish species such as Nile tilapia (*Oreochromis niloticus*), tiger puffer (*Takifugu rubripes*) and spotted scat (*Scatophagus argus*) (Tao et al., 2013; Jiang et al., 2017; Yan et al., 2018; He et al., 2019; Chen et al., 2021). Other CYP components are also involved in the steroid hormone biosynthesis pathway and are required for sex steroid production in various teleost species including rainbow trout, European sea bass (*Dicentrachus labrax*) and spotted scat (Liu et al., 2000; Socorro et al., 2007; Blázquez et al., 2017; He et al., 2019; Meng et al., 2020; Nyuji et al., 2020). Our study identified eight steroid-metabolizing enzymes with *cyp1a1, cyp11b, cyp26c1* and *cyp21a2* overexpressed in testes, especially the first two showing male-specific expression; while *cyp19a1, cyp11a, cyp26a1* and *cyp17a2* highly upregulated in the ovaries. *Cyp1a1* has a role in E2 metabolism in mammals, and is suggested to be primarily responsible for E2 metabolism in zebrafish (Scornaienchi et al., 2010; Sun et al., 2018). High expression of *cyp1a1* was observed in the ovaries of Japanese sardine (Nyuji et al., 2020) and stinging catfish (Chaube et al., 2021), suggesting its critical role in the complex regulation of FOM and ovulation. However, *cyp1a1* is overexpressed in the testes of Olive Flounder (*Paralichthys olivaceus*) (Fan et al., 2014). P450 11β-hydroxylase (*cyp11b*), a key enzyme for the synthesis of 11-ketotestosterone (11-KT) in testes, is a potent masculinising steroid in fish species such as honeycomb groupers (*Epinephelus merra*) (Bhandari et al., 2006; Wang and Orban, 2007). For example, the expression of *cyp11b* was observed to be either male-specific or comparatively higher in the testes of European sea bass (Socorro et al., 2007), rainbow trout (Liu et al., 2000), tiger puffer (Yan et al., 2018), Spot-fin porcupinefish (Chen et al., 2021) and Japanese flounder (Meng et al., 2020). In tambaqui (*C. macropomum*) however, *cyp1a1* was upregulated in females (Lobo et al., 2020). Therefore, the CYP members identified in this study may play potential roles in the development of gonads and participate in regulating reproductive functions (e.g., synthesis of steroid hormones) of cobia.

Moreover, two members of *wnt* family including *wnt1* and *wnt4b* were also exclusively expressed in the testes of cobia. Th *wnt* family has been linked to testicular development and suppression of developmental pathways in ovaries (Tevosian and Manuylov, 2008). One of the best characterized components in wnt/b-catenin signaling pathway, *wnt4*, is involved in the mice testes determination pathway *via* acting with the testis-determining gene *Sry* to initiate proper testes differentiation. *wnt4* is also known as one of the few key ovarian-determining genes in mammals (Vainio et al., 1999). This indicated that *wnt4* can have a specific, but distinct role in both male and female gonad development (Jeays-Ward et al., 2004). Different from mammals, fish possess multiple subtypes of *wnt4* (*wnt4a1, wnt4a2* and *wnt4b*) due to teleost-specific whole genome duplication. The three different *wnt4* subtypes were observed to be either strongly expressed in ovarian tissue or in the early stage of testes for several studied fish species including medaka (Li et al., 2012), orange-spotted grouper (Chen et al., 2015), black seabream (Wu and Chang, 2009), spotted scat (Chen et al., 2016), half-smooth tongue sole (Hu et al., 2014b), olive flounder (Chen et al., 2016) and rainbow trout (Nicol et al., 2012). These results showed that fish do not display an ovary-predominant *wnt4* expression profile during early gonadal differentiation. Similar to *wnt4*, the *wnt1* was observed in the testes of mice (Erickson et al., 1993), however, this gene is also known to act in the WNT/β-catenin signaling pathway, to the membrane of oocytes and early preimplantation embryos in mammals (Harwood et al., 2008). In fish, a previous study in lake sturgeon (*Acipenser fulvescens*) showed *wnt1* was more highly expressed in females compared to males (Hale et al., 2010). The male-specific expression pattern of *wnt1* and *wnt4b* presented in our study, suggests that they may be necessary to trigger testicular differentiation in male cobia, and thus could provide an important clue for gonad differentiation of the species. Therefore, further underlying functional analysis of these genes and their regulatory network should be conducted.

In addition, DEGs such as *vasa*, *zar1*, three *zps,* nine *spatas*, and *piwil* involving germ cell development, gametogenesis and gamete maturation, were also in the gonad of cobia in the current study. A recent publication (Ma et al., 2022) showed that the *vasa* gene appeared to be specifically expressed in the testes and ovaries of cobia and mainly expressed in germ cells by qPCR and chromogenic *in situ* hybridization (CISH) analysis. Zona pellucida plays a protective role in fish oocytes, and is important in sperm binding (Litscher and Wassarman, 2018). In the present study, *zp1, zp3* and *zp4* showed higher expression levels in females than in males, suggesting these genes may also play critical roles in folliculogenesis and reproduction in cobia. Overall, these identified gonocyte-specific genes may be helpful for investigating the control mechanisms during oogenesis and spermatogenesis in cobia. Furthermore, according to functional prediction and classification, the identified gonadal differentially expressed genes of cobia were significantly enriched in eleven reproductive related pathways including the Wnt signaling pathway, MAPK signaling pathway and TGF-beta signaling pathway, which have all play important roles in the development of ovarian and testicular functions. The pathway enrichment analyses for the ovary- and testis-biased genes provide insights into the molecular landscape underlying the functions of ovaries and testes. The comparison of ovarian and testicular transcriptomes offers a precious resource for further investigation of the genetic basis of sex determination, sex differentiation and sexual size dimorphism of cobia. In the future, our current study may be complemented with mRNA, miRNA and lncRNA sequencing for undifferentiated gonads and differentiated gonads of immature males and immature females to have more comprehensive studies for investigation of the sex determination and sex differentiation of the species.

## Conclusion

This study compared ovary and testis transcriptomes of cobia, identifying a set of DEGs and pathways known to be involved in gonadal development, gametogenesis and physiological function. This is the first comparative gonad transcriptomic study in cobia, providing significant information to enrich the genetic resources of the species. Furthermore, the valuable information on sex-associated genes could further facilitate exploration of the molecular mechanisms of gonad development and gametogenesis in cobia. Our findings can also provide a reference for future research on the mechanism of sexual growth dimorphism and sex control breeding of monosex female cobia populations in commercial farming.

## Data Availability

The datasets presented in this study can be found in online repositories. The names of the repository/repositories and accession number(s) can be found below: https://www.ncbi.nlm.nih.gov/genbank/, PRJNA853560.
